# Optimized workflow for pentaspline PFA and its impact on PVI durability: evidence from systematic remapping

**DOI:** 10.1093/europace/euag118

**Published:** 2026-05-13

**Authors:** Daniel Rodríguez-Muñoz, Lucía Cobarro Gálvez, Ez Alddin Rajjoub Al-Mahdi, Isabel López-Alacid, Javier Ramos Jiménez, Álvaro Marco del Castillo, Luis Borrego Bernabé, Martín Negreira Caamaño, Lorena Gómez Burgueño, Fernando Arribas Ynsaurriaga, Rafael Salguero-Bodes

**Affiliations:** Cardiology Department, Hospital Universitario 12 de Octubre, Avda. de Córdoba s/n, Madrid 28041, Spain; Instituto de Investigación Hospital 12 de Octubre (imas12), Avda. de Córdoba s/n, Madrid 28041, Spain; Cardiology Department, Hospital Universitario 12 de Octubre, Avda. de Córdoba s/n, Madrid 28041, Spain; Instituto de Investigación Hospital 12 de Octubre (imas12), Avda. de Córdoba s/n, Madrid 28041, Spain; Cardiology Department, Hospital Universitario 12 de Octubre, Avda. de Córdoba s/n, Madrid 28041, Spain; Instituto de Investigación Hospital 12 de Octubre (imas12), Avda. de Córdoba s/n, Madrid 28041, Spain; Cardiology Department, Hospital Universitario 12 de Octubre, Avda. de Córdoba s/n, Madrid 28041, Spain; Instituto de Investigación Hospital 12 de Octubre (imas12), Avda. de Córdoba s/n, Madrid 28041, Spain; Cardiology Department, Hospital Universitario 12 de Octubre, Avda. de Córdoba s/n, Madrid 28041, Spain; Instituto de Investigación Hospital 12 de Octubre (imas12), Avda. de Córdoba s/n, Madrid 28041, Spain; Cardiology Department, Hospital Universitario 12 de Octubre, Avda. de Córdoba s/n, Madrid 28041, Spain; Instituto de Investigación Hospital 12 de Octubre (imas12), Avda. de Córdoba s/n, Madrid 28041, Spain; Cardiology Department, Hospital Universitario 12 de Octubre, Avda. de Córdoba s/n, Madrid 28041, Spain; Instituto de Investigación Hospital 12 de Octubre (imas12), Avda. de Córdoba s/n, Madrid 28041, Spain; Cardiology Department, Hospital Universitario 12 de Octubre, Avda. de Córdoba s/n, Madrid 28041, Spain; Instituto de Investigación Hospital 12 de Octubre (imas12), Avda. de Córdoba s/n, Madrid 28041, Spain; Cardiology Department, Hospital Universitario de Getafe, Getafe, Spain; Cardiology Department, Hospital Universitario 12 de Octubre, Avda. de Córdoba s/n, Madrid 28041, Spain; Instituto de Investigación Hospital 12 de Octubre (imas12), Avda. de Córdoba s/n, Madrid 28041, Spain; Cardiology Department, Hospital Universitario 12 de Octubre, Avda. de Córdoba s/n, Madrid 28041, Spain; Instituto de Investigación Hospital 12 de Octubre (imas12), Avda. de Córdoba s/n, Madrid 28041, Spain; Department of Medicine, Faculty of Medicine, Universidad Complutense, Madrid, Spain; Centro de Investigación Biomédica en Red de Enfermedades CardioVasculares (CIBERCV), Instituto de Salud Carlos III (ISCIII), Madrid, Spain; Cardiology Department, Hospital Universitario 12 de Octubre, Avda. de Córdoba s/n, Madrid 28041, Spain; Instituto de Investigación Hospital 12 de Octubre (imas12), Avda. de Córdoba s/n, Madrid 28041, Spain; Department of Medicine, Faculty of Medicine, Universidad Complutense, Madrid, Spain; Centro de Investigación Biomédica en Red de Enfermedades CardioVasculares (CIBERCV), Instituto de Salud Carlos III (ISCIII), Madrid, Spain

## Abstract

**Aims:**

Real-world series report substantial rates of pulmonary vein (PV) reconnection following index procedure with a pentaspline pulsed-field ablation (P-PFA) catheter. The contrast with initial durability data suggests this may be due to limitations in procedural workflow.

**Methods and results:**

We conducted a single-centre, prospective study including consecutive patients undergoing first-time P-PFA pulmonary vein isolation (PVI), subsequently scheduled for systematic invasive remapping ≥30 days after the index procedure. Workflow was progressively refined in three phases, aiming at ensuring adequate tissue contact and based on previous remapping findings. The primary endpoint was PVI durability, per vein and per patient. Of 118 enrolled patients, 94 (Phase 1:*n* = 30; Phase 2:*n* = 34; Phase 3:*n* = 30) underwent systematic remapping, where 13, 10, and 1 reconnected PVs were observed in Phases 1, 2, and 3, respectively. This resulted in per-vein durability of 89.8%, 92.9%, and 99.2% (*P* = 0.005) and per-patient durability of 60.0%, 76.5%, and 96.7% (*P* = 0.004). Mean total PFA applications increased from 36.8 ± 5.2 in Phase 1 to 52.1 ± 8.0 and 58.7 ± 15.1 in Phases 2 and 3, respectively (*P* < 0.001). Median procedure time rose from 43 (39–50) minutes in Phase 1 to 46 (42–56) minutes in Phase 2 and 69 (61–77) minutes in Phase 3 (*P* < 0.001), with significant parallel increases in LA dwell time (*P* < 0.001) across phases. No major or minor complications occurred during index or remapping procedures.

**Conclusion:**

Progressive workflow refinements for pentaspline *P*-PFA markedly improved PVI durability to nearly 100%, supporting the concept that PV reconnection is predominantly technique-dependent rather than intrinsic to catheter or waveform design.

## Graphical Abstract

**Graphical Abstract:**
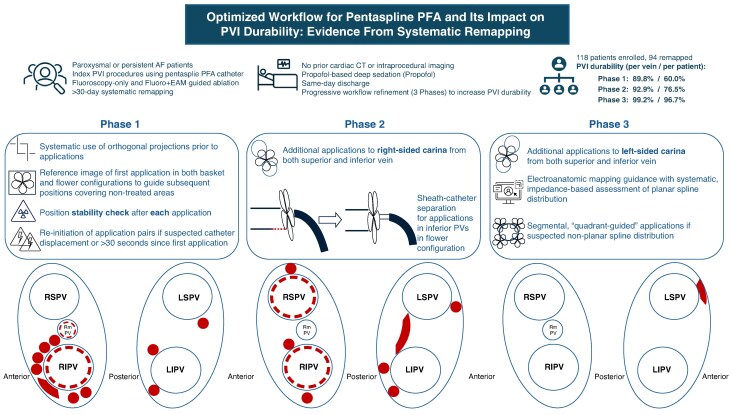

## Introduction

Atrial fibrillation (AF) represents the most prevalent sustained cardiac arrhythmia and is associated with significant morbidity, mortality, and impaired quality of life. The rising incidence of AF, together with accumulating evidence supporting the effectiveness of pulmonary vein isolation (PVI), has prompted recent clinical guidelines to endorse catheter ablation as a first-line therapeutic option in selected patients. As a result, procedural volumes have increased substantially in recent years.^1–5^

In this context, the pentaspline pulsed-field ablation (P-PFA) catheter offers increased safety, at least comparable clinical efficacy, and shorter procedure times compared with thermal ablation for AF ablation.^6–8^ While radiofrequency and cryoballoon ablation have shown high rates of pulmonary vein reconnection in patients undergoing repeat electrophysiological evaluation for AF recurrence,^9,10^ early evaluations showed a remarkably high rate of durable PVI with P-PFA.^11^ These observations suggested that PFA could mitigate the historical limitations of thermal ablation related to late PV reconnection and positioned the technology as a potentially robust solution for long-term lesion durability. In line with this, PFA has been proposed as a first-line energy source for PVI.^12,13^

However, real-world experience, including data from large volume centres beyond the initial learning curve, has revealed a non-negligible incidence of reconnections, comparable to those historically observed with thermal ablation and persisting even in the most contemporary datasets.^14–16^ These findings highlight the need to optimize procedural strategies to maximize lesion durability while maintaining procedural efficiency. Although fluoroscopic guidance remains standard practice in many centres, the adoption of intracardiac echocardiography (ICE)^17^ and electroanatomic mapping (EAM)^18^ to support device navigation is increasing, with the potential to improve lesion quality at the expense of greater complexity and resource utilization.

We hypothesized that PV reconnections after P-PFA are not primarily driven by limitations in catheter design or waveform efficacy but rather by suboptimal catheter–tissue contact and deployment technique—factors that are amenable to systematic workflow optimization. Accordingly, the aim of this study was to evaluate PVI durability in patients undergoing systematic remapping after PFA, assessing the impact of a set of workflow measures applied in a standardized, systematic manner.

## Methods

### Study design

We performed a single-centre, prospective, observational study enrolling consecutive patients with paroxysmal or persistent AF undergoing PVI with a P-PFA catheter. Procedures were guided either by fluoroscopy alone (Phases 1 and 2) or by fluoroscopy combined with the electroanatomic mapping with the Faraview module on Opal HDx (Marlborough, MA, USA) navigation system. All patients were planned for invasive remapping at least 30 days after the index ablation, regardless of recurrence status.

The study was registered at clinicaltrials.gov (ID: NCT06706518) and conducted at a tertiary university hospital, with systematic remapping performed as part of the clinical study, and not routine practice. All patients provided written informed consent, and the study was approved by the institutional review board and conforms to the Declaration of Helsinki. Patient data were processed in compliance with the EU General Data Protection Regulation (2016/679) and applicable national and institutional data protection policies.

### Study population

Patients aged > 18 years old with a diagnosis of paroxysmal or persistent AF and a clinical indication to undergo PVI were included. Exclusion criteria were prior PVI or left atrial (LA) linear ablation, severe frailty (Clinical Fraily Scale of 7 or higher) or life expectancy <1 year, unwillingness or inability to provide informed consent, any indication for ablation at sites beyond PVI, contraindication or intolerance to heparin, presence of LA thrombus, congenital heart disease, and pregnancy, ongoing or planned in the following 6 months. Eligible patients were provided with detailed information regarding the study's objectives and the necessity for additional, study-mandated invasive remapping as part of the consent process.

The study aimed to enrol 30 remapped patients per phase to ensure that outcomes were both generalizable and indicative of each workflow's performance. The predefined criteria for study termination included achieving a PVI durability of at least 95% per vein or 85% per patient, with a maximum of four phases established by successive workflow iterations.

### Endpoints

The primary efficacy endpoint was PVI durability, assessed as a dual endpoint:

1. per-vein durability, defined as the proportion of electrically isolated PVs at systematic remapping;
2. per-patient durability, defined as the proportion of patients with all PVs isolated at remapping.

Secondary endpoints included total fluoroscopy time, recurrence of AF or any atrial tachyarrhythmia (AT) during follow-up, the number of applications delivered during the index procedure, total procedure duration, left atrial dwell time, acute PVI success rate, and total radiation dose.

The primary safety endpoint was a composite of major adverse events (AEs) occurring within 30 days after the index procedure, including cardiac perforation, cardiac tamponade, stroke or transient ischaemic attack, peripheral thromboembolic events, vascular complications requiring intervention, myocardial infarction, or death. Adverse events occurring within 30 days after the remapping procedure were also systematically documented.

#### Procedural workflow

All procedures were performed by operators having performed over 100 PVI procedures using the P-PFA catheter prior to the initiation of Phase 1. All patients with persistent AF and those with paroxysmal AF and a CHA_2_DS_2_-VA score >1, underwent transoesophageal echocardiography within 72 h prior to the procedure to exclude intracardiac thrombus, with no other cardiac imaging techniques performed. Procedures were carried out under uninterrupted oral anticoagulation and deep sedation with propofol and fentanyl. Unfractionated heparin was administered immediately before or after transseptal puncture to maintain an activated clotting time ≥300 s. A 1 mg bolus of atropine was given to all patients to prevent vagally mediated bradycardia, except for those with a cardiac implantable electronic device capable of ventricular pacing.

Single femoral venous access was obtained under ultrasound guidance. Transseptal puncture was performed under fluoroscopic visualization using an SL-0 sheath (Abbott, Chicago, IL) and a BRK-1-XS Brockenbrough needle (Abbott, Chicago, IL, USA). A J-tip guidewire was advanced into the LA to facilitate exchange of the SL-0 sheath for a deflectable Faradrive™ sheath (Boston Scientific, Marlborough, MA, USA). A 31-mm Farawave™ (Phases 1 and 2) or Farawave Nav™ (Phase 3) catheter (Boston Scientific, Marlborough, MA, USA) was then introduced through the sheath and positioned in the left superior pulmonary vein (LSPV) over a Starter-J guidewire (Boston Scientific, Marlborough, MA, USA). Subsequent procedural steps are detailed below. Left common pulmonary veins (LCPV), when present, were treated as separate veins.

Acute PVI was confirmed by demonstrating both entrance and exit block using the P-PFA catheter, in line with current evidence.^19^

At the end of the procedure, haemostasis was achieved using a figure-of-eight suture and pressure dressing, both removed 4–6 h post-procedure. Oral anticoagulation was resumed 4 h after ablation and maintained for at least 2 months, or indefinitely according to individual thromboembolic risk as determined by the CHA_2_DS_2_-VA score. Antiarrhythmic drugs were not routinely prescribed and were discontinued 2 months post-ablation when previously in use. Per institutional protocol, patients were discharged 5 h after the procedure in the absence of complications.

### Phase 1. Systematic, double-projection, fluoroscopy-only approach

The 31-mm Farawave catheter was navigated exclusively by fluoroscopic guidance following a systematic protocol using two complementary projections—left anterior oblique (LAO) 30° and right anterior oblique (RAO) 20°, with an additional 20°caudal tilt for superior PVs—prior to each set of PFA applications. For each vein, the goal of the ipsilateral fluoroscopic projection was to ensure contact and, in a flower configuration, to prevent splines from being inadvertently placed inside the vein. The goal of the contralateral projection was to ensure proper sheath-catheter-guidewire alignment (see Supplementary material online, *Figure S1*) and to ensure applications were properly delivered on the whole circumference of the ostium and antrum of each vein. To ensure this, a reference image of the first application was acquired and displayed on a reference screen. This served to check catheter stability between the first and second application of each pair, as well as to guide spline positioning in subsequent applications, aiming to cover all inter-spline spaces observed in the initial position. This was applied both to basket and flower configurations. Pulsed field was delivered per manufacturer’s standard settings, that is, 2 kV bipolar, biphasic applications over 2.5 s for each application. Additionally, applications were repeated if, between the first and second applications of each pair, catheter displacement had occurred, or if >30 s had elapsed.

### Phase 2. Systematic, double-projection, fluoroscopy -only optimized approach

This phase maintained the systematic approach described in Phase 1, adding two refinements to address the potential causes for the gaps identified in chronic remapping. First, to reduce gaps at the right-sided carina and anterior-superior aspect of the right inferior PV, applications were delivered to the right-sided carina from both the superior—applying posterior torque and adding sheath deflection—and inferior—anterior torque and reducing sheath deflection—PVs. Second, to reduce gaps at the inferior aspect of the inferior PVs, catheter advance to contact the PV antrum in flower configuration was performed by progressing the catheter itself and using the sheath in a 90° angle to provide direction (see Supplementary material online, *Figure S2*), instead of progressing the sheath to push the flower directly towards the tissue, as this was interpreted to potentially facilitate displacement from the interior rim of inferior PVs.

### Phase 3. Systematic, double-projection, fluoroscopy with integration of impedance values from electroanatomic navigation system

This phase maintained the systematic approach described in Phases 1 and 2 but incorporated two more steps in the procedure. First, systematic additional applications towards left-side carina from both superior and inferior left PVs. Second, EAM guidance was added with the Faraview module on Opal HDx. Specifically, impedance-value-based depiction of the electrically active electrodes was assessed prior to applications in flower configuration to ensure that none were inadvertently placed inside the vein (see Supplementary material online, *Figure S3*). If this was detected, catheter was repositioned to achieve a homogeneous planar distribution of all electrodes. If repositioning was unsuccessful, additional dedicated applications to the untreated antral segments were delivered.

### Invasive remapping

Systematic invasive remapping was scheduled at least 30 days after the index procedure, regardless of arrhythmia recurrence status. Periprocedural management, including sedation protocol, vascular access, and anticoagulation strategy, was identical to that used during the index procedure. Left atrial access was initially attempted through the transeptal puncture performed in the index procedure, by progressing the guidewire into the LA. If unsuccessful, transeptal puncture was repeated with the same workflow as in the index procedure. High-density bipolar voltage maps were acquired using an EAM System—OPAL™ (Boston Scientific, Marlborough, MA, USA), CARTO® 3 (Biosense Webster, Irvine, CA, USA), or EnSite X™ (Abbott, Chicago, IL, USA)—to assess the primary efficacy endpoint.

Durable PVI was defined as bidirectional conduction block, confirmed by the absence of PV potentials and by exit block during pacing within the vein at 8.0 V and 2.0 ms output. When necessary, a deflectable diagnostic catheter was positioned in the superior vena cava or the left atrial appendage to differentiate far-field electrograms. Identified conduction gaps were targeted with a contact-force-sensing, irrigated ablation catheter.

### Clinical follow-up

Routine clinical follow-up was carried out to assess secondary endpoints, including AF or AT recurrence, as well as post-procedural adverse events. Standardized outpatient evaluations and 24-hour ECG monitoring were performed at 3, 6, and 12 months post-ablation.

### Statistical analysis

Continuous variables were expressed as mean ± standard deviation or median with interquartile range (IQR), according to data distribution assessed by the Shapiro–Wilk test. Comparisons across groups were performed using one-way analysis of variance (ANOVA) for normally distributed variables or the Kruskal–Wallis test for non-normally distributed variables. When ANOVA indicated statistically significant differences, *post hoc* pairwise comparisons between groups were performed with Bonferroni correction for multiple testing. Categorical variables were summarized as counts and percentages and compared using the χ^2^ test or Fisher’s exact test, as appropriate.

Given the unequal follow-up duration across treatment phases, limiting direct comparability of arrhythmia recurrence rates, a complementary phase-specific analysis was performed, focusing on the first 180 days of follow-up.^20^ An 8-week blanking period was applied. Freedom from atrial tachyarrhythmia recurrence at 180 days was assessed using the Kaplan–Meier method and the log-rank test.

The primary efficacy endpoint—PVI durability—was evaluated both per vein and per patient, and between-group comparisons were performed using χ^2^ or Fisher’s exact tests. Recurrence of AF or other atrial tachyarrhythmias (AT) during follow-up was analysed as a binary outcome.

All statistical tests were two-tailed, and a *P* value < 0.05 was considered statistically significant. Statistical analyses were performed using Stata/IC version 17.0 (StataCorp LLC, College Station, TX, USA).

## Results

### Baseline patient characteristic

**Figure 1:**
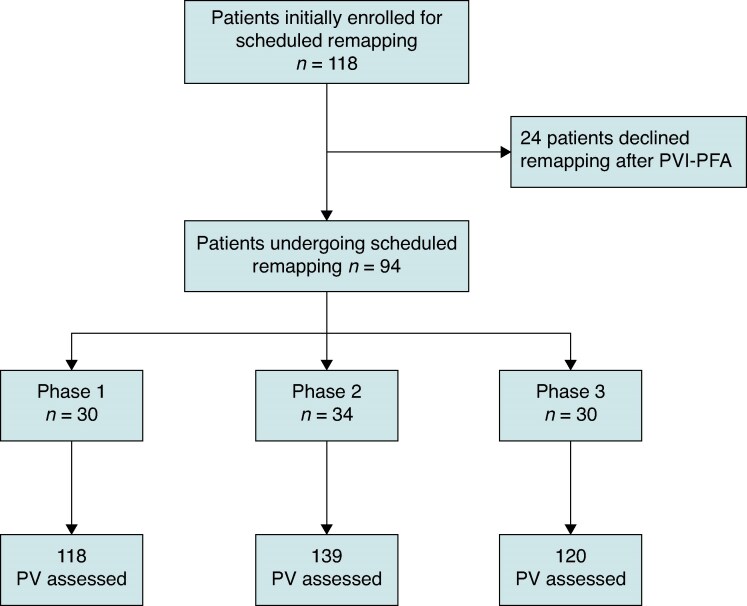
Study flowchart. A total of 118 patients undergoing index PFA-PVI were initially enrolled for scheduled remapping (Phase 1: 44; Phase 2: 38; Phase 3: 36). Of these, all 118 patients were contacted for remapping. Of them, 24 declined the remapping procedure (Phase 1: 14; Phase 2: 4; Phase 3: 6), due to having reconsidered their willingness to undergo a second intervention (22 patients), complaints about groin pain after the index procedure (1 patient), and limited availability due to work schedule (1 patient). This resulted in a final cohort of 94 patients who underwent remapping (Phase 1: 30; Phase 2: 34; Phase 3: 30). A total of 377 PVs (118, 139, and 120 in Phases 1, 2, and 3, respectively) were reassessed at remapping.

A total of 118 patients were initially included in the study. Of these, 24 (14 patients in Phase 1, 4 in Phase 2, and 6 in Phase 3) declined participation when contacted for the remapping procedure, leading to a total of 94 undergoing invasive remapping, corresponding to procedures performed in Phase 1 (*n* = 30), Phase 2 (*n* = 34), and Phase 3 (*n* = 30) (*Figure 1*). Overall, 34.0% were female, the mean age was 64.9 ± 10.4 years, and the mean body mass index (BMI) was 28.6 ± 4.2 kg/m^2^. Hypertension (66.0%) and diabetes mellitus (23.4%) were the most prevalent comorbidities. The median CHA_2_DS_2_-VA score was 2 (IQR, 1–3). Cardiomyopathy was present in 39.4% of patients (11.7% ischaemic, 3.2% non-ischaemic dilated, and 24.4% other aetiologies).

Echocardiographic evaluation showed that left ventricular ejection fraction was normal (>50%) in 85.6%, mildly reduced (41–50%) in 6.7%, moderately reduced (36–40%) in 3.3%, and severely reduced (<35%) in 4.4% of patients. The mean indexed LA volume was 42.6 ± 14.4 mL/m^2^. QRS duration exceeded 120 ms in 14.9% of patients, including one patient with ventricular pacing.

Regarding AF type, 45.7% had paroxysmal and 54.3% persistent AF. The median time from AF diagnosis to PFA was 355.5 days (IQR 179–1359). At baseline, 12.8% of patients were receiving flecainide and 14.9% amiodarone.

Baseline characteristics were largely comparable across groups (*Table 1*).

**Table 1:** Baseline characteristics across the three procedural groups

Variable	Phase 1 (*n* = 30)	Phase 2 (*n* = 34)	Phase 3 (*n* = 30)	*P*-value
Age (years), mean ± SD	67.6 (9.3)	61.6 (11.3)	66.1 (9.5)	0.05
Female sex, *n* (%)	11 (36.7)	8 (23.5)	13 (43.3)	0.23
BMI (kg/m^2^), mean ± SD	29.2 (3.7)	28.5 (3.7)	28.2 (5.1)	0.67
Hypertension, *n* (%)	18 (60.0)	22 (64.7)	22 (73.3)	0.54
Diabetes mellitus, *n* (%)	11 (36.7)	5 (14.7)	6 (20.0)	0.10
Dyslipidaemia, *n* (%)	10 (33.3)	8 (23.5)	7 (23.3)	0.15
Obstructive sleep apnoea, *n* (%)	4 (13.3)	4 (11.8)	1 (3.3)	0.35
Chronic kidney disease, *n* (%)	3 (10.0)	3 (8.8)	2 (6.7)	0.9
Previous stroke/TIA, *n* (%)	3 (10.0)	1 (2.9)	4 (13.3)	0.31
CHA_2_DS_2_-VA score, median (IQR)	3 (1-3)	2 (1–3)	2 (1–3)	0.11
Cardiomyopathy, *n* (%)	14 (46.7)	14 (41.2)	9 (30.0)	0.15
– Ischaemic, *n* (%)	5 (16.7)	3 (8.8)	3 (10.0)	
– Non-ischaemic, *n* (%)	3 (10.0)	0	0	
– Other	6 (20.0)	11 (32.4)	6 (20.0)	
Normal LVEF (%)	23 (76.7)	29 (85.3)	25 (83.3)	0.53
Left atrial index volume (mL/m^2^), mean ± SD	43.5 (16.4)	43.0 (14.8)	40.4 (9.7)	0.80
Persistent AF, *n* (%)	19 (63.3)	18 (52.9)	14 (46.7)	0.42
Time from AF diagnosis to PFA < 1 year, *n* (%)	14 (46.7)	21 (61.8)	13 (43.3)	0.28
Antiarrhythmic drug use	8 (26.7)	8 (23.5)	10 (33.3)	0.71
– Amiodarone, *n* (%)	5 (16.7)	5 (14.7)	4 (13.3)	
– Flecainide, *n* (%)	3 (10.0)	3 (8.8)	6 (20.0)	
Oral anticoagulation				
– Vitamin K antagonist, *n* (%)	5 (16.7)	8 (23.5)	3 (10.0)	0.35
– Direct oral anticoagulant, *n* (%)	24 (80.0)	26 (76.5)	25 (83.3)	0.60

Data are presented as mean ± standard deviation for continuous variables, as median with interquartile range for non-normally distributed continuous variables, and as number (percentage) for categorical variables. BMI, body mass index; LVEF, left ventricular ejection fraction; TIA, transient ischaemic attack.

### PFA procedural characteristics

Procedural characteristics of the PFA procedures are summarized in *Table 2*. All procedures were performed under deep sedation with continuous propofol infusion and intravenous fentanyl analgesia. A total of 377 PVs were targeted during the index PVI procedures (Phase 1 = 118; Phase 2 = 139; Phase 3 = 120). Acute PVI was successfully achieved in all patients.

**Table 2:** PFA procedural characteristics

Variable	Phase 1 (*n* = 30)	Phase 2 (*n* = 34)	Phase 3 (*n* = 30)	*P*-value
Total PFA applications (mean ± SD)	36.8 ± 5.2	52.1 ± 8.0	58.7 ± 15.1	<0.001
− LSPV-Basket [median (IQR)]	4 (4–4)	4 (4–5)	4 (4–8)	
− LSPV-Flower [median (IQR)]	4 (4–5)	6 (5–8)	8 (6–12)	
− LIPV-Basket [median (IQR)]	4 (4–4)	4 (4–6)	5 (4–6)	
− LIPV-Flower [median (IQR)]	4 (4–4)	6 (4–8)	6 (6–8)	
− RSPV-Basket [median (IQR)]	4 (4–5)	5 (4–6)	6 (4–8)	
− RSPV-Flower [median (IQR)]	4 (4–6)	10 (8–13)	12 (8–15)	
− RIPV-Basket [median (IQR)]	4 (4–4)	4.5 (4–6)	4 (4–6)	
− RIPV-Flower [median (IQR)]	4 (4–5)	8 (6–10)	9 (8–11)	
Procedure time, min [median (IQR)]	43 (39–50)	46 (42–56)	69 (61–77)	<0.001
LA dwell time, min [median (IQR)]	25 (23–28)	32 (28–39)	54 (46–59)	<0.001
Fluoroscopy time, min [median (IQR)]	3.9 (2.8–7.2)	4.9 (4.3–6.3)	8.2 (6.0–9.5)	<0.001

Data are shown as mean ± standard deviation for normally distributed continuous variables, as median with interquartile range for non-normally distributed continuous variables, and as number (percentage) for categorical variables. *P*-values correspond to global comparisons across the three phases. The table additionally reports the number of PFA applications delivered *per vein* and *per catheter configuration* (basket and flower). PFA, pulsed field ablation; LA, left atrial; IQR, interquartile range; SD, standard deviation; LSPV, left superior pulmonary vein; LIPV, left inferior pulmonary vein; RSPV, right superior pulmonary vein; RIPV, right inferior pulmonary vein.

The mean total number of PFA applications per procedure was 36.8 ± 5.3 in Phase 1, 52.1 ± 8.0 in Phase 2, and 58.7 ± 15.1 in Phase 3 (*P* < 0.001).

The median total procedure time was 52 min (43–65), with phase-specific values of 43 min (39–50), 46 min (42–56), and 69 min (61–77) in Phases 1, 2, and 3, respectively (*P* < 0.001). The median LA dwell time was 25 min (23–28), 32 min (28–39), and 54 min (46–59) in Phases 1, 2, and 3, respectively (*P* < 0.001). Pairwise comparisons with Bonferroni correction showed that Phase 3 was associated with a significantly longer procedure time compared with Phases 1 and 2 (*P* < 0.001), as well as a significantly longer LA dwell time compared with Phases 1 and 2 (*P* < 0.001). No significant differences were observed between Phases 1 and 2 for either procedure or LA dwell time.

Fluoroscopy time differed across phases. Median fluoroscopy time was 3.9 min (2.8–7.2) in Phase 1, 4.9 min (4.3–6.3) in Phase 2, and 8.2 min (6.0–9.5) in Phase 3 (*P* < 0.001). After Bonferroni correction, fluoroscopy time was significantly higher in Phase 3 compared with Phase 2 (*P* < 0.001), while no significant differences were observed between Phase 1 and the other phases.

**Figure 2:**
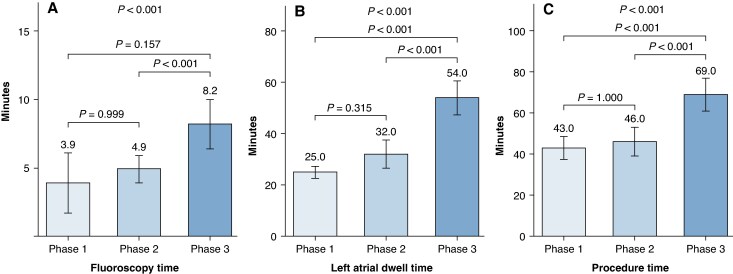
Procedural, fluoroscopy, and left atrial dwell times across study phases. Median values for fluoroscopy time (*A*), left atrial (LA) dwell time (*B*), and total procedure time (*C*) are shown for Phases 1, 2, and 3. Error bars represent interquartile ranges. Progressive increases in procedural and LA dwell times reflect the adoption of more comprehensive ablation workflows in later phases, whereas fluoroscopy time increased only in Phase 3 following the integration of electroanatomic navigation.

Procedural time, LA dwell time, and fluoroscopy time across study phases is summarized in *Figure 2*.

### Remapping results

Median time from the index procedure to the invasive evaluation of chronic PVI durability was 178 days (110–235) in Phase 1, 42 days (34–92) in Phase 2, and 38 days (34–52) in Phase 3 (*P* < 0.001), with longer time in Phase 1 due to administrative issues including insurance and availability of the materials supplied for remapping.

A total of 12, 10, and 1 reconnected PVs were identified in phases 1, 2, and 3, respectively. Per-vein isolation durability was 89.8%, 92.8%, and 99.2% in phases 1, 2, and 3, respectively (*P* = 0.005) (*Table 3*). Pairwise comparisons showed a significantly higher per-vein durability in Phase 3 compared with Phase 1. Per-patient isolation durability also increased significantly across phases, from 60.0% in Phase 1% to 76.5% in Phase 2% and 96.7% in Phase 3 (*P* = 0.004). Pairwise comparisons showed that Phase 3 was associated with a significantly higher per-patient durability compared with Phases 1 and 2, whereas no significant difference was observed between Phases 1 and 2.

**Table 3:** Remapping outcomes by procedural phase

Variable	Phase 1 (*n* = 30)	Phase 2 (*n* = 34)	Phase 3 (*n* = 30)	*P*-value
Total PVs assessed, *n*	118	139	120	—
Time to remapping, days [median (IQR)]	178 (110–235)	42 (34–92)	38 (33–52)	<0.001
Reconnected PVs, *n*	12	10	1	—
PVI durability per-vein, %	89.8	92.8	99.2	0.005
PVI durability per-patient (%)	60.0	76.5	96.7	0.004
Patients with ≥ 2 reconnection, *n* (%)	0	2	0	—

Values are presented as counts or median with interquartile range, as appropriate. Per-vein reconnection rate was calculated as the proportion of pulmonary veins demonstrating electrical reconnection at systematic remapping. PV, pulmonary vein.

**Figure 3:**
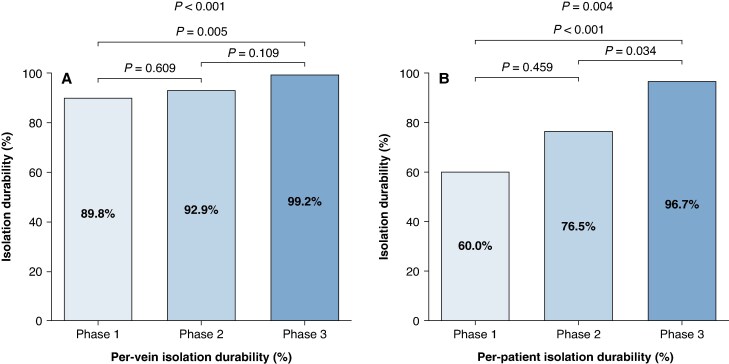
Durability of pulmonary vein isolation across study phases. (*A*) *Per-vein isolation durability*, calculated as the proportion of pulmonary veins without reconnection at remapping, significantly increased across phases: 89.8% in Phase 1, 92.8% in Phase 2, and 99.2% in Phase 3. (*B*) *Per-patient isolation durability*, defined as the proportion of patients with all PVs durably isolated showed increasing success rate: 60.0% in Phase 1, 76.5% in Phase 2, and 96.7% in Phase 3.

**Figure 4:**
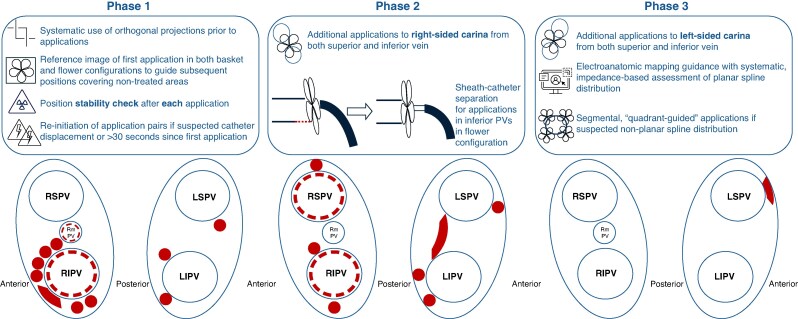
Schematic representation of the actions implemented in each phase and gap location in remapping. LIPV = left inferior pulmonary vein; LSPV = left superior pulmonary vein; RIPV = right inferior pulmonary vein; RSPV = right superior pulmonary vein.

Two patients in Phase 2 had two reconnected PV each. *Figure 3* summarizes remapping results. *Figure 4* shows a schematic distribution of gap locations.

Regarding anatomical variations, an LCPV was identified at remapping in three patients in Phase 1, 2 patients in Phase 2, and 5 patients in Phase 3; all were found to be durably isolated. As for right middle pulmonary veins, 1 was identified in Phase 1 (not isolated, as it had not been targeted during the index procedure), 3 in Phase 2 (all isolated), and 4 in Phase 3 (all isolated).

### Safety outcomes

No procedural complications, major or minor, were observed during either the index PFA procedures or the remapping studies. There were no vascular, pericardial, thromboembolic, phrenic nerve, oesophageal, or sedation-related adverse events.

### Clinical follow-up

The median follow-up duration differed significantly across phases, with 675.5 days (598–765) in Phase 1, 367 days (277–534) in Phase 2, and 218.5 days (189–259) in Phase 3 (*P* < 0.001). During overall follow-up, atrial arrhythmia recurrence was documented in 8 patients (26.7%) among those treated in Phase 1, consisting of AF in 5 patients, macro–re-entrant AT in 2 patients, and both AF and AT in 1 patient. Among those treated in Phase 2, recurrence occurred in 6 (14.7%) patients, consisting of AF in 4 patients, macro–re-entrant AT in 1 patient, and both AF and AT in 1 patient. Only one AF recurrence was documented in patients treated during Phase 3. The presence of at least one reconnected PV was significantly higher among patients with atrial arrhythmia recurrence (44.4% vs. 23.4%, *P* = 0.02).

**Figure 5:**
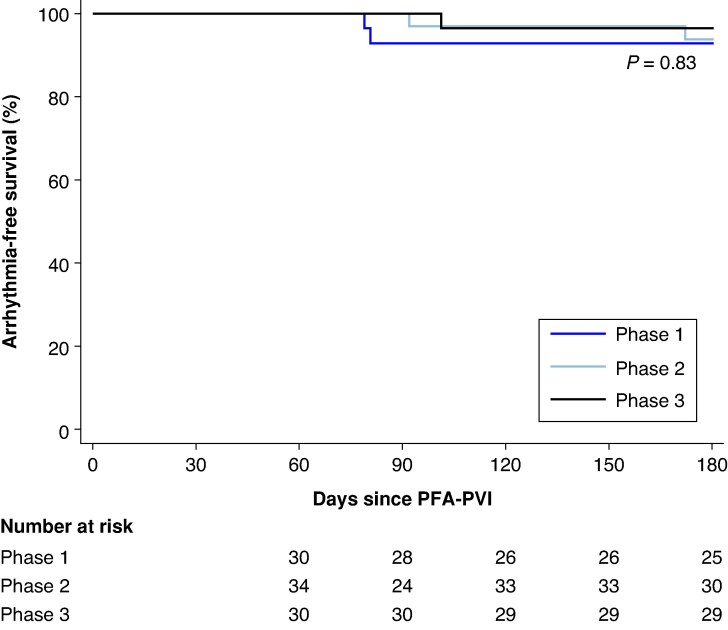
Kaplan–Meier curves of arrhythmia-free survival according to treatment phase. PFA-PVI: pulsed field ablation pulmonary vein isolation.

Given the unequal follow-up duration across phases, a complementary analysis focusing on the first 180 days of follow-up was performed. During this period, atrial tachyarrhythmia recurrence occurred in 4 patients (13.3%) treated in Phase 1, 2 patients (5.9%) treated in Phase 2, and 1 patient treated in Phase 3 (*Figure 5*) (log-rank *P* = 0.83).

## Discussion

Our study shows that specific refinements in PFA procedural workflow have a meaningful impact on long-term lesion durability. Across the three phases, the progressive integration of specific workflow modifications translated into a marked reduction in PV reconnections, achieving near-complete durable isolation in Phase 3. These observations support the notion that the primary determinant of durable PVI with the pentaspline catheter is not the intrinsic performance of the device or waveform, but rather the consistency and quality of catheter–tissue engagement throughout the procedure.

Invasive systematic remapping is the gold standard for assessing PVI durability. This study provides the most extensive systematic remapping analysis to date, performed with the commercially available Pentaspline catheter. Our findings are particularly relevant in light of recent real-world evidence, given that large contemporary registries have shown that durable isolation is far from guaranteed in routine clinical practice. In the EU-PORIA registry, 72% of PVs were found to be durably isolated and complete durable PVI was observed in only 38% of patients undergoing redo procedures.^14^ In the MANIFEST-REDO study, per-vein reconnections ranged from 27% to 32%, with just 45% of patients exhibiting durable isolation of all PVs.^15^ Importantly, both registries assessed durability exclusively in patients with clinically indicated repeat procedures. As a result, reported reconnection rates reflect only those patients with arrhythmia recurrence rather than PVI durability rates of the full population, likely to be higher given the known association of AF recurrence with PV reconnection, also observed in our study. Among the 8 patients with AT or AF recurrence in our study, which may be considered a more comparable cohort to those in EU-PORIA and MANIFEST REDO, no PV reconnections were observed in 55.6% of patients, which is superior to the averages in these studies and close to the best performing centre in EU-PORIA.^13^

The natural evolution of any new ablation technology involves early variability, a learning curve, and progressive workflow optimization; P-PFA is no exception. A growing body of evidence supports the need to refine P-PFA techniques to improve PVI outcomes. Recent technique-oriented publications describe how to perform PVI with the P-PFA catheter and consistently emphasize the importance of technical execution—particularly catheter coaxiality and adequate catheter–tissue contact—as key determinants of durable lesions.^21,22^ Similarly, different groups have identified common areas where PV reconnections typically occur, such as the carina between right-sided PVs, and proposed possible causes and workflow modifications to prevent them.^14,23^ However, these recommendations remain largely based on observational data and expert opinion, often derived from heterogeneous cohorts of redo procedures performed for arrhythmia recurrence.

Beyond fluoroscopy, complementary tools such as intracardiac echocardiography (ICE) and 3D EAM can provide real-time visualization of catheter–tissue interaction and may enhance lesion quality. In a cohort of patients undergoing redo procedures, Natale et al. recently reported reconnection in 10.3% of patients with ICE guidance compared with 59.2% when fluoroscopy alone was used, concluding that ICE-confirmed catheter–tissue contact significantly improves success and reduces reconnection rates in PFA.^17^ Nonetheless, ICE substantially increases procedural costs and remains unevenly available worldwide. In our study, a standardized and systematically applied set of workflow refinements yielded higher lesion durability even without the use of ICE, particularly in Phase 3, when EAM-guided impedance-value assessment of adequate spline placement at the PV antrum was integrated in the procedure.

A complementary observation from our study is that procedural, LA dwell time, and fluoroscopy time increased progressively across phases. This pattern contrasts with several previous reports and trials describing a remarkably short learning curve for the P-PFA system, with rapid reductions in procedure duration and radiation exposure even among operators early in their experience.^14,16^ However, rather than reflecting inefficiency, the longer times observed in our study were the direct consequence of intentionally adding specific technical measures aimed at maximizing catheter–tissue contact and lesion quality. This highlights a critical distinction between simply ‘learning to use P-PFA’ and ‘learning to optimize P-PFA for durable lesion formation’, a nuance not captured in prior large real-world datasets. In addition, the number of applications also increased significantly, exceeding 60 applications in a non-negligible proportion of patients in Phase 3. Although significant haemolysis has been reported in procedures exceeding 50 applications,^19^ improving tissue contact has also been reported to reduce its occurrence.^20^ Notably, in our cohort, there were no immediate or late post-procedural adverse events that could be linked to haemolysis. However, acute kidney injury has been reported in procedures with numbers of applications previously considered ‘safe’, so the threshold for haemolysis-safe P-PFA procedures is still unclear and dependent on multiple factors.^24^

Whether PVI durability outcomes in Phase 1 are an adequate benchmark, representative of real-life use of this catheter, especially without systematic ICE guidance, could be debated. Regarding procedural characteristics, Phase 1 total procedure and LA dwell times were slightly shorter than those of other studies. However, they were, on average, only 3 and 7 min shorter, respectively, than those in Phase 2, which achieved 76.5% per patient durable PVI. This would suggest that outcomes of Phase 1 procedures were not poorer due to a particularly careless approach but are probably representative of what can be expected from a non-refined workflow. Fluoroscopy time was notably shorter than that reported in other studies due to a strategy developed at our centre, validated for cryoballoon PVI and later adopted for PFA devices. Our earlier research showed clinical outcomes remained consistent despite significant reductions in fluoroscopy time.^25^ Similarly, fluoroscopy time increased by 1.0 min between Phases 1 and 2, while the number of applications increased by 15.3. This would suggest that too short fluoroscopy times were not a major determinant of the outcomes observed in Phase 1.

Finally, the absence of major or minor complications across all phases highlights the favourable safety profile of this refined workflow, with procedural risk even lower than that previously reported.^11,16,26^

## Limitations

Our study has some limitations. First, it was conducted at a single centre, which may limit generalizability despite its prospective design and intention to clearly define the changes in workflow. Second, procedural refinements were introduced cumulatively across phases, preventing the determination of the individual contribution of each workflow modification. Consequently, this study cannot give an answer to the question of how much of the increase in effectiveness is attributable to each change in workflow, including the addition of electroanatomic navigation and careful catheter positioning, suggested by the increase in LA dwell time and number of applications. Third, the follow-up period differed among phases, limiting the long-term comparability of clinical outcomes. However, prior work^20^ has shown that more than half of first atrial tachyarrhythmia recurrences occur within the first 3 months after rhythm control interventions, with nearly 80% by 6 months and over 90% by 9 months, and that earlier recurrence is associated with greater long-term AF burden. This supports the use of a 180-day analysis window to capture most clinically meaningful recurrences. Besides, although invasive remapping is the gold standard for assessing PVI durability, and the relation between it and atrial arrhythmia recurrence is well established,^27–29^ impact of the different workflows in clinical outcomes cannot be established from these data. Fourth, given the nature of the study, the effect of the learning curve, although presumed minor in operators having performed more than 100 P-PFA cases before the initiation of the study, cannot be excluded. Fifth, time to remap differs across phases, although per study design all remapping procedures were performed more than 30 days after the index procedure, when PFA lesions have shown to be mature and fully representative of its long-term characteristics.^26–29^

## Conclusion

In this prospective, phase-based evaluation of a progressively optimized P-PFA workflow for PVI, the systematic introduction of targeted procedural refinements led to a marked and incremental improvement in PVI durability, culminating in almost 100% durable isolation in Phase 3. These gains were accompanied by longer fluoroscopy exposure, LA dwell time, and procedure duration, and a higher number of applications. Taken together, our findings support the hypothesis that durable PVI with PFA depends primarily on deployment technique rather than on advanced imaging tools or inherent catheter or waveform design. We conclude that an optimized workflow can deliver near-complete durable PVI with a favourable safety profile.

## Supplementary Material

euag118_Supplementary_Data

## Data Availability

The data underlying this article are available in the article. Further data will be shared on reasonable request to the corresponding author.
