# Supplementary figures and images for: Optimized workflow for pentaspline PFA and its impact on PVI durability: evidence from systematic remapping
Source: Europace. 2026 May 13;28(6):euag118. doi: 10.1093/europace/euag118 (PMC13229260; doi:10.1093/europace/euag118)

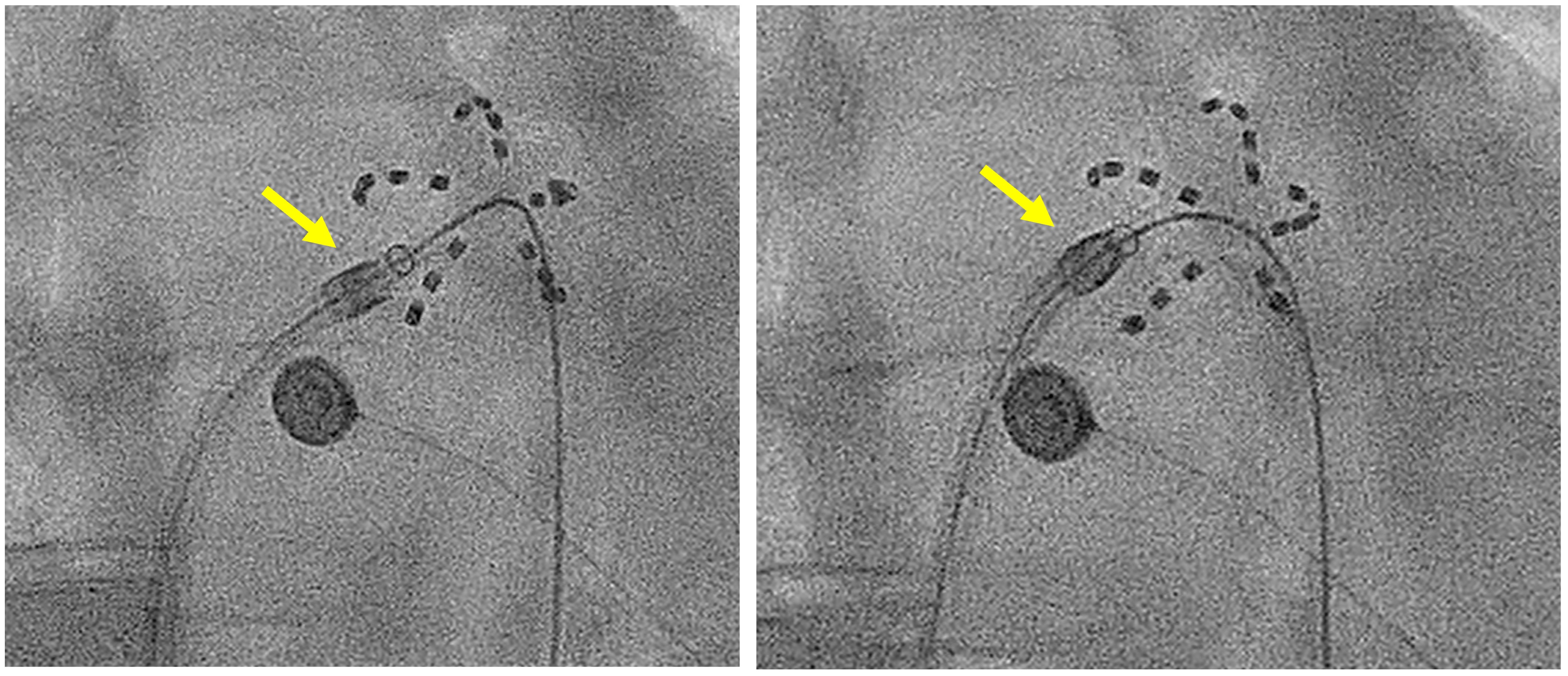

Supplement: euag118_Supplementary_Data [file euag118_supplementary_data.zip › Sup Fig 1 - Contralat project to assess alignment.jpg]

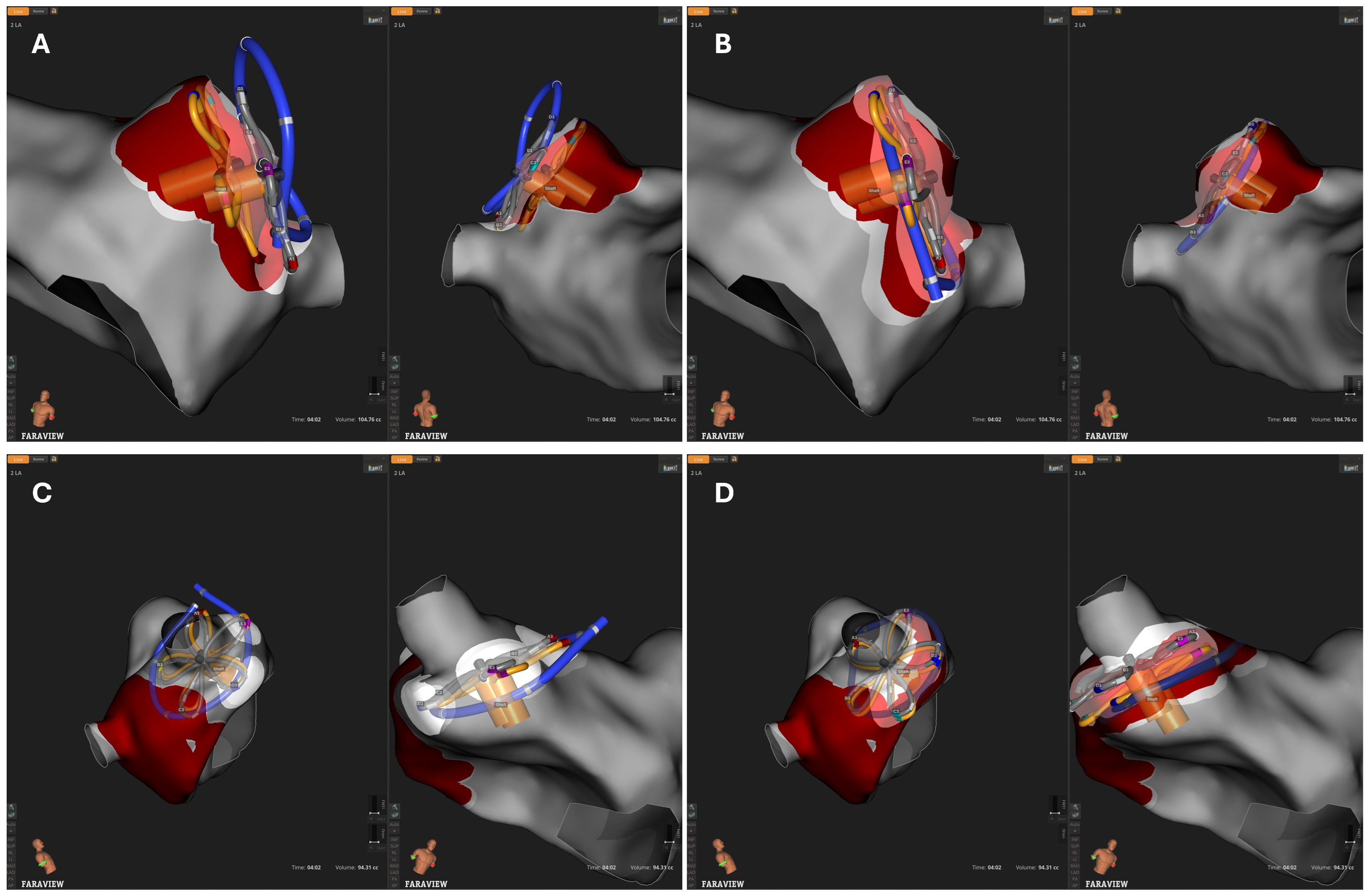

Supplement: euag118_Supplementary_Data [file euag118_supplementary_data.zip › Sup Fig 3 - Imped. planar distribution.jpg]

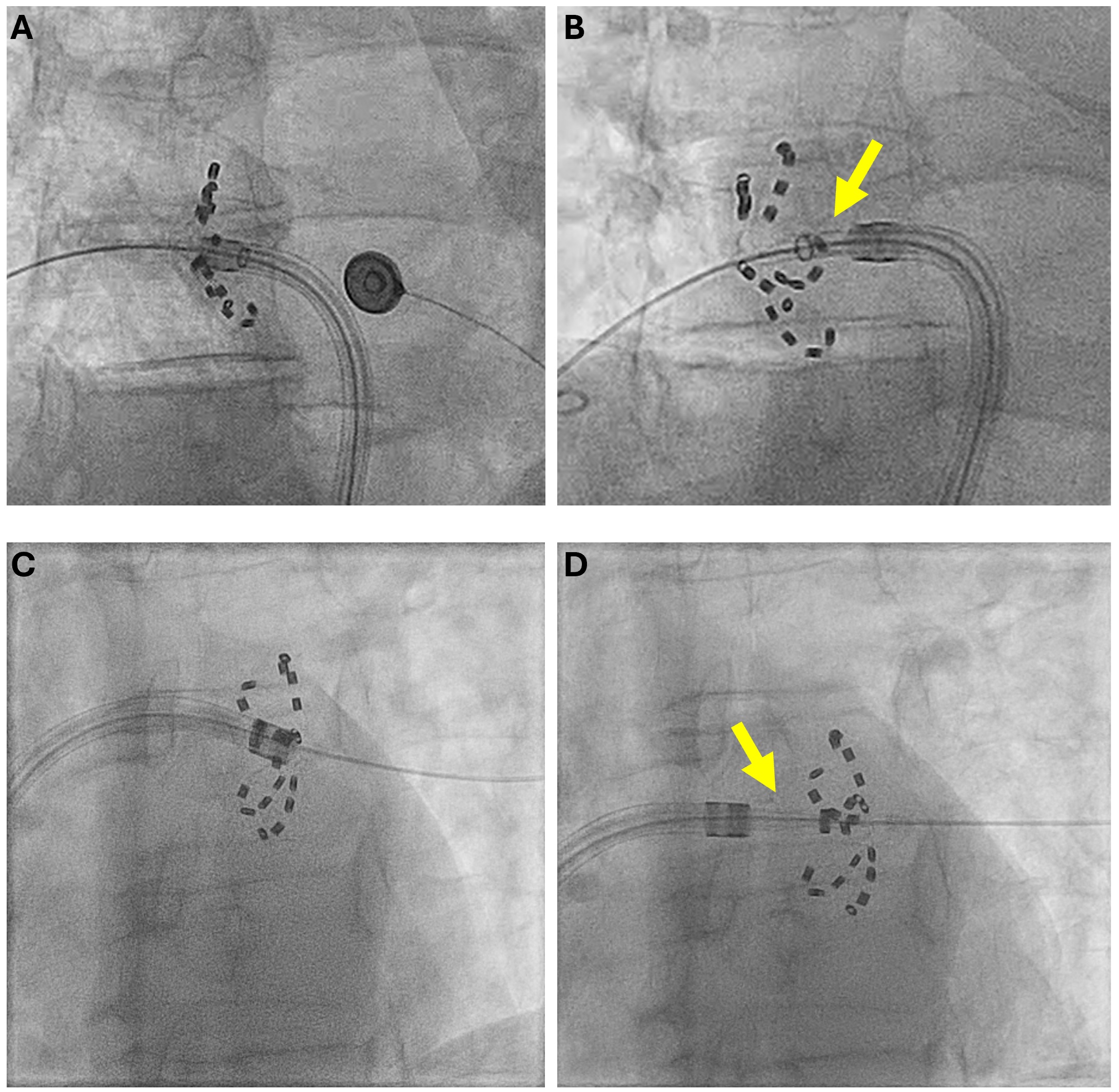

Supplement: euag118_Supplementary_Data [file euag118_supplementary_data.zip › Suppl Figure 2 - Approach to inferior PVs.jpg]
